# Polar Decomposition of Jones Matrix and Mueller Matrix of Coherent Rayleigh Backscattering in Single-Mode Fibers

**DOI:** 10.3390/s24061760

**Published:** 2024-03-08

**Authors:** Hui Dong, Hailiang Zhang, Dora Juan Juan Hu

**Affiliations:** Institute for Infocomm Research (I2R), Agency for Science, Technology and Research (A*STAR), 1 Fusionopolis Way, #21-01, Connexis South Tower, Singapore 138632, Singapore; zhang_hailiang@i2r.a-star.edu.sg (H.Z.); jjhu@i2r.a-star.edu.sg (D.J.J.H.)

**Keywords:** optical polarization, fiber optics, Rayleigh backscattering, Jones matrix, Mueller matrix, phase-sensitive optical time-domain reflectometry

## Abstract

The Jones matrix and the Mueller matrix of the coherent Rayleigh backscattering (RB) in single-mode fibers (SMFs) have been derived recently. It has been shown that both matrices depict two polarization effects—birefringence and polarization-dependent loss (PDL)—although the SMF under investigation is purely birefringent, having no PDL. In this paper, we aim to perform a theoretical analysis of both matrices using polar decomposition. The derived sub-Jones/Mueller matrices, representing birefringence and PDL, respectively, can be used to investigate the polarization properties of the coherent RB. As an application of the theoretical results, we use the derived formulas to investigate the polarization properties of the optical signals in phase-sensitive optical time-domain reflectometry (φ-OTDR). For the first time, to our knowledge, by using the derived birefringence–Jones matrix, the common optical phase of the optical signal in φ-OTDR is obtained as the function of the forward phase and birefringence distributions. By using the derived PDL–Mueller matrix, the optical intensity of the optical signal in φ-OTDR is obtained as the function of the forward phase and birefringence distributions as well as the input state of polarization (SOP). Further theoretical predictions show that, in φ-OTDR, the common optical phase depends on only the local birefringence in the first half of the fiber section, which is occupied by the sensing pulse, irrelevant of the input SOP. However, the intensity of the φ-OTDR signal is not a local parameter, which depends on the input SOP and the birefringence distribution along the entire fiber section before the optical pulse. Moreover, the PDL measured in φ-OTDR is theoretically proven to be a local parameter, which is determined by the local birefringence and local optical phase distributions.

## 1. Introduction

In recent years, phase-sensitive optical time-domain reflectometry (φ-OTDR) has attracted more and more attention from various research communities due to the overwhelming advantages over traditional sensors. φ-OTDR was proposed firstly for distributed intrusion sensing [1,2]. Since then, it has been used for structure health monitoring [3], partial discharge locating and detecting [4], railway monitoring [5], downhole measurement [6], traffic monitoring [7], pipeline leakage detection and localization [8], earthquake detection [9], rising bubble measurement [10], and many other applications [11]. In particular, by using submarine fiber-optic cables, φ-OTDR is used for hydroacoustic signal detection [12], submarine structural characterization [13], coastal safety [14], water column and sediment monitoring [15], near-field target localization in shallow water [16], etc. In addition to the commonly used telecommunication fiber-optic cables, φ-OTDR technology has also been verified in datacenter bidirectional fiber links [17], submarine power cables [18], and backscattering-enhanced fiber [2].

The sensing optical signal in φ-OTDR originates from the coherent Rayleigh backscattering (RB) in single-mode fibers (SMFs). Therefore, the properties of the coherent RB in SMFs are of great importance for understanding the principles and improving the performances of φ-OTDR. The polarization properties of the coherent RB play an important role in φ-OTDR because the φ-OTDR signal is determined by the optical phase and the state of polarization (SOP) of the coherent RB. However, only preliminary works have been reported to study the polarization-related issues in φ-OTDR. Yixin Zhang et al. found that local birefringence changes in SMF lead to the generation of polarization-dependent noise and failure to identify multipoint vibration events [19]. Sterenn Guerrier et al. presented a simple and incomplete calculation of the round-trip Jones matrix in φ-OTDR [20,21]. Since the detailed result is not presented, the polarization effects are not considered in the reported physical models of φ-OTDR [22,23]. To understand the polarization properties of the coherent RB when the incident light is perfectly coherent, we recently derived the Jones matrix and the Mueller matrix that govern the polarization properties of the coherent RB [24]. With these matrices, we theoretically predicted and then experimentally verified the properties of the SOP and the degree of polarization (DOP) of the coherently superposed RB light [24]. However, the polarization properties of the optical signals detected in φ-OTDR have not yet been investigated in detail, especially the relationship between the distributions of optical phase and fiber birefringence, and the common optical phase and optical power (intensity), which are two optical signals usually detected in φ-OTDR.

This paper is a continuation of the previous works reported in [24]. In this paper, further theoretical calculation is performed using polar decomposition methods to decompose the known Jones matrix and Mueller matrix into sub-matrices that describe birefringence and PDL, respectively. For the first time, to our knowledge, with the derived birefringence–Jones matrix, the common optical phase can be explicitly expressed as the function of the distributions of fiber birefringence and optical phase. With the derived PDL–Mueller matrix, the optical power can also be depicted by the input SOP and the distributions of fiber birefringence and optical phase.

This paper is organized as follows: In Section 2, the polar decomposition of the Jones matrix is performed, then the common optical phase of the coherent RB is derived. In Section 3, the polar decomposition of the Mueller matrix is conducted. In Section 4, the polarization properties of the intensity and the common optical phase in φ-OTDR are analyzed based on the formulas obtained in Section 2 and Section 3.

## 2. Polar Decomposition of the Jones Matrix of the Coherent RB

**Figure 1:**
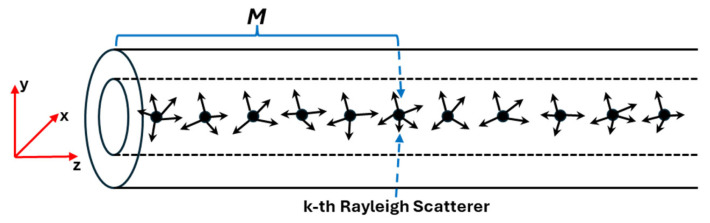
The SMF under investigation. There are N Rayleigh scatterers distributed along the z direction of the SMF. The forward Mueller matrix describing the fiber section from the input end to the *k*-th Rayleigh scatterer is M.

Assuming that there are N Rayleigh scatterers in the SMF under investigation, as illustrated in Figure 1, the Jones matrix $J_{RTS}$, which governs the polarization properties of the coherent RB, has been derived as [24]

(1) $$J_{RTS}=\left[\begin{array}{cc} t_{1} & t_{2}\\ t_{2} & t_{3} \end{array}\right]$$

The matrix elements in Equation (1) are [24]

(2) $$\left\{\begin{array}{l} t_{1}=q_{8}+p_{9}+i\left(q_{9}-p_{8}\right)\\ t_{2}=-q_{7}+ip_{7}\\ t_{3}=-q_{8}+p_{9}+i\left(q_{9}+p_{8}\right) \end{array}\right.$$

where $i=\sqrt{-1}$. The notations in Equation (2) are defined as [24]

(3) $$\left\{\begin{array}{l} p_{j}=\sum_{k=1}^{N}c_{k}m_{jk}\cos2\varphi_{k}\\ q_{j}=\sum_{k=1}^{N}c_{k}m_{jk}\sin2\varphi_{k}\\ \vec{p}=\left[p_{7}p_{8}p_{9}\right],\;\vec{q}=\left[q_{7}q_{8}q_{9}\right] \end{array}\right.j=7,8,9$$

where the subscript k stands for the *k*-th Rayleigh scatterer in the SMF; $c_{k}=\sqrt{\gamma_{k}}e^{-\rho_{k}}$, $\rho_{k}$ is the fiber attenuation, and $\gamma_{k}$ is the RB reflection coefficient; $\varphi_{k}$ is the optical phase. The items $m_{jk},\;j=7,8,9$ are the matrix elements of the forward Mueller matrix M at the fourth row and the second, third and fourth column, respectively [24]. The details of M are described in Appendix A.

It has been demonstrated that any Jones matrix can be interpreted as a cascade of a retarder (optical element with birefringence) and a diattenuator (optical element with PDL) [25]. Therefore, $J_{RTS}$ can be decomposed as [25,26]

(4) $$J_{RTS}=UH$$

where U is a unitary matrix satisfying $U^{+}U=I$, “+” denotes the conjugate transpose and I is the identity matrix; H is a Hermitian matrix satisfying $H^{+}=H$. Therefore, U represents birefringence and H represents PDL. By using the polar decomposition method proposed in [26], we calculate U and H as follows:

Due to $H^{+}=H$, the Hermitian matrix H can be expanded in terms of four basic Pauli spin matrices [26]

(5) $$H=\sum_{j=0}^{3}h_{j}\sigma_{j}=\left[\begin{array}{cc} h_{0}+h_{1} & h_{2}-ih_{3}\\ h_{2}+ih_{3} & h_{0}-h_{1} \end{array}\right]$$

where four Pauli spin matrices are defined as $\sigma_{0}=\left[\begin{array}{cc} 1 & 0\\ 0 & 1 \end{array}\right]$, $\sigma_{1}=\left[\begin{array}{cc} 1 & 0\\ 0 & -1 \end{array}\right]$, $\sigma_{2}=\left[\begin{array}{cc} 0 & 1\\ 1 & 0 \end{array}\right]$, and $\sigma_{3}=\left[\begin{array}{cc} 0 & -i\\ i & 0 \end{array}\right]$ in this paper; $h_{j},\;j=0,1,2,3$ are the four expansion coefficients. The square of H is calculated as

(6) $$H^{2}=\left({h_{0}}^{2}+h^{2}\right)\sigma_{0}+2h_{0}\sum_{j=1}^{3}h_{j}\sigma_{j}$$

where $h^{2}=\sum_{j=1}^{3}{h_{j}}^{2}$. On the other hand, Equation (4) leads to

(7) $$W={J_{RTS}}^{+}J_{RTS}=H^{+}U^{+}UH=H^{+}IH=H^{2}=\sum_{j=0}^{3}w_{j}\sigma_{j}$$

A comparison between Equations (6) and (7) leads to

(8) $$H^{2}=\left({h_{0}}^{2}+h^{2}\right)\sigma_{0}+2h_{0}\sum_{j=1}^{3}h_{j}\sigma_{j}={J_{RTS}}^{+}J_{RTS}=\left[\begin{array}{cc} t_{1}t_{1}^{*}+t_{2}t_{2}^{*} & t_{1}^{*}t_{2}+t_{2}^{*}t_{3}\\ t_{1}t_{2}^{*}+t_{2}t_{3}^{*} & t_{2}t_{2}^{*}+t_{3}t_{3}^{*} \end{array}\right]$$

where the superscript “*” denotes the conjugate of a complex number. From Equation (2), the matrix elements in Equation (8) are computed as

(9) $$\left\{\begin{array}{l} t_{1}t_{1}^{*}+t_{2}t_{2}^{*}=p_{7}^{2}+p_{8}^{2}+p_{9}^{2}+q_{7}^{2}+q_{8}^{2}+q_{9}^{2}+2\left(q_{8}p_{9}-p_{8}q_{9}\right)\\ t_{2}t_{2}^{*}+t_{3}t_{3}^{*}=p_{7}^{2}+p_{8}^{2}+p_{9}^{2}+q_{7}^{2}+q_{8}^{2}+q_{9}^{2}-2\left(q_{8}p_{9}-p_{8}q_{9}\right)\\ t_{1}^{*}t_{2}+t_{2}^{*}t_{3}=2\left(p_{7}q_{9}-q_{7}p_{9}\right)-2i\left(q_{7}p_{8}-p_{7}q_{8}\right)\\ t_{1}t_{2}^{*}+t_{2}t_{3}^{*}=2\left(p_{7}q_{9}-q_{7}p_{9}\right)+2i\left(q_{7}p_{8}-p_{7}q_{8}\right) \end{array}\right.$$

The terms $p_{7}^{2}+p_{8}^{2}+p_{9}^{2}+q_{7}^{2}+q_{8}^{2}+q_{9}^{2}$, $2\left(q_{8}p_{9}-p_{8}q_{9}\right)$, $2\left(p_{7}q_{9}-q_{7}p_{9}\right)$, and $2\left(q_{7}p_{8}-p_{7}q_{8}\right)$ in Equation (9) can be expressed as $w_{j},j=0,1,2,3$, respectively, and further calculated by substituting Equation (3) into Equation (9). The results are as follows:

(10) $$\left\{\begin{array}{ll} w_{0} & ={\left|\vec{p}\right|}^{2}+{\left|\vec{q}\right|}^{2}=p_{7}^{2}+p_{8}^{2}+p_{9}^{2}+q_{7}^{2}+q_{8}^{2}+q_{9}^{2}\\ & =\sum_{k,l=1}^{N}c_{k}c_{l}\left({\hat{m}}_{k}\cdot {\hat{m}}_{l}\right)\cos2∆\varphi_{kl}\;\\ & =\sum_{k=1}^{N}c_{k}^{2}+\sum_{k,l=1;k\neq l}^{N}c_{k}c_{l}\left({\hat{m}}_{k}\cdot {\hat{m}}_{l}\right)\cos2∆\varphi_{kl}\;\\ w_{1} & =2\left(q_{8}p_{9}-p_{8}q_{9}\right)=\sum_{k,l=1}^{N}c_{k}c_{l}\left(m_{8k}m_{9l}-m_{8l}m_{9k}\right)\sin2∆\varphi_{kl}\;\\ w_{2} & =2\left(p_{7}q_{9}-q_{7}p_{9}\right)=\sum_{k,l=1}^{N}c_{k}c_{l}\left(m_{9k}m_{7l}-m_{9l}m_{7k}\right)\sin2∆\varphi_{kl}\\ w_{3} & =2\left(q_{7}p_{8}-p_{7}q_{8}\right)=\sum_{k,l=1}^{N}c_{k}c_{l}\left(m_{7k}m_{8l}-m_{7l}m_{8k}\right)\sin2∆\varphi_{kl} \end{array}\right.$$

where $∆\varphi_{kl}=\varphi_{k}-\varphi_{l}$, and ${\hat{m}}_{k}=\left[m_{7k}\;m_{8k}\;m_{9k}\right]$. Based on Equation (A3) and Equation (A5) in Appendix A, $\left|{\hat{m}}_{k}\right|=1$. The properties of $w_{j},\;j=0,1,2,3$ are further investigated and shown in Appendix B.

From Equations (8)–(10), it has

(11) $$\left({h_{0}}^{2}+h^{2}\right)\sigma_{0}+2h_{0}\sum_{j=1}^{3}h_{j}\sigma_{j}\;\;\;\;\;\;\;\;\;\;\;\;\;\;\;\;\;\;\;\;\;\;\;\;\;\;\;\;\;\;\;\;\;\;\;\;\;\;\;\;\;\;\;\;\;\;\;\;\;\;\;\;\;\;\;\;\;\;\;\;\;\;\;\;\;\;\;\;\;\;\;\;\;\;\;\;\;\;\;\;\;\;\;\;\;\;\;\;\;\;\;\;\;\;\;\;=\left[\begin{array}{cc} {h_{0}}^{2}+h^{2}+2h_{0}h_{1} & 2h_{0}\left(h_{2}-ih_{3}\right)\\ 2h_{0}\left(h_{2}+ih_{3}\right) & {h_{0}}^{2}+h^{2}-2h_{0}h_{1} \end{array}\right]=\left[\begin{array}{cc} w_{0}+w_{1} & w_{2}-iw_{3}\\ w_{2}+iw_{3} & w_{0}-w_{1} \end{array}\right]$$

Equation (11) brings four equations relating to $h_{j},\;j=0,1,2,3$, with $w_{j},\;j=0,1,2,3$. Then, $h_{j},\;j=0,1,2,3$ can be expressed in terms of $w_{j},\;j=0,1,2,3$ as

(12) $$\left\{\begin{array}{l} h_{0}=\frac{\sqrt{w_{0}+w}+\sqrt{w_{0}-w}}{2}\\ h_{j}=\frac{w_{j}}{2h_{0}},\;\;j=1,2,3\\ h=\frac{\sqrt{w_{0}+w}-\sqrt{w_{0}-w}}{2}\\ w=\sqrt{\sum_{j=1}^{3}{w_{j}}^{2}}=2\left|\vec{q}\times \vec{p}\right| \end{array}\right.$$

From Equation (5), the inverse matrix of H is calculated as

(13) $$H^{-1}=\frac{\left[\begin{array}{cc} h_{0}-h_{1} & -\left(h_{2}-ih_{3}\right)\\ -\left(h_{2}+ih_{3}\right) & h_{0}+h_{1} \end{array}\right]}{h_{0}^{2}-h^{2}}$$

Then, from Equation (4), it has

(14) $$U=J_{RTS}H^{-1}=\frac{\left[\begin{array}{cc} t_{1}\left(h_{0}-h_{1}\right)-t_{2}\left(h_{2}+ih_{3}\right) & t_{2}\left(h_{0}+h_{1}\right)-t_{1}\left(h_{2}-ih_{3}\right)\\ t_{2}\left(h_{0}-h_{1}\right)-t_{3}\left(h_{2}+ih_{3}\right) & t_{3}\left(h_{0}+h_{1}\right)-t_{2}\left(h_{2}-ih_{3}\right) \end{array}\right]}{h_{0}^{2}-h^{2}}$$

Eventually, the Jones matrix $J_{RTS}$ has been decomposed into a unitary matrix U in the form of Equation (14) and a Hermitian matrix H in the form of Equation (5). The matrix elements in U and H have been expressed in terms of the optical phase φ and the forward Muller matrix elements $m_{j},\;j=7,8,9$ through Equations (2), (3), (10), and (12).

Since the unitary matrix U represents the birefringence effect, it should be expressed in the general form of [27]

(15) $$U=e^{i\varepsilon }\left[\begin{array}{cc} u_{1} & u_{2}\\ -u_{2}^{*} & u_{1}^{*} \end{array}\right]$$

where ε is the common optical phase, and $u_{1}u_{1}^{*}+u_{2}u_{2}^{*}=1$. Hence, the determinant of U should be

(16) $$det\left(U\right)=e^{2i\varepsilon }\left(u_{1}u_{1}^{*}+u_{2}u_{2}^{*}\right)=e^{2i\varepsilon }$$

On the other hand, from Equation (14), the determinant of the birefringence sub-Jones matrix U is computed as

(17) $$det\left(U\right)=\frac{t_{1}t_{3}-t_{2}^{2}}{h_{0}^{2}-h^{2}}=e^{i\tau }$$

where

(18) $$tan\tau =\frac{2\left(p_{7}q_{7}+p_{8}q_{8}+p_{9}q_{9}\right)}{p_{7}^{2}-q_{7}^{2}+p_{8}^{2}-q_{8}^{2}+p_{9}^{2}-q_{9}^{2}}=\frac{\sum_{k,l=1}^{N}c_{k}c_{l}\left({\hat{m}}_{k}\cdot {\hat{m}}_{l}\right)\sin2\left(\varphi_{k}+\varphi_{l}\right)}{\sum_{k,l=1}^{N}c_{k}c_{l}\left({\hat{m}}_{k}\cdot {\hat{m}}_{l}\right)\cos2\left(\varphi_{k}+\varphi_{l}\right)}$$

The detailed steps to calculate Equations (17) and (18) are shown in Appendix C. From Equations (15) and (16), it has

(19) $$\varepsilon =\frac{\tau }{2}\;$$

This means that the common optical phase of the coherent RB should be ε=τ/2, which is the optical signal measured in the phase-based φ-OTDR for quantitative strain measurement [28]. Equation (18) presents how ε is determined by the optical phase distribution $\varphi_{k}$ and the birefringence distribution ${\hat{m}}_{k}$ in the SMF. To the best of our knowledge, it is the first time that such a formula is obtained when the fiber birefringence is taken into consideration. When the fiber birefringence is not considered, it can be demonstrated, as shown in Appendix D, that Equation (18) aligns with the known equation without birefringence terms.

## 3. Polar Decomposition of the Mueller Matrix of the Coherent RB

The corresponding Mueller matrix $M_{RTS}$ of the coherent RB has already been obtained as [24]

(20) $$M_{RTS}=\left[\begin{array}{cccc} w_{0} & w_{1} & w_{2} & w_{3}\\ w_{1} & v_{11} & v_{12} & v_{13}\\ w_{2} & v_{12} & v_{22} & v_{23}\\ -w_{3} & -v_{13} & {-v}_{23} & {-v}_{33} \end{array}\right]$$

The matrix elements $w_{0}$, $w_{1}$, $w_{2}$, and $w_{3}$ have already been defined in Equation (10). The rest of the matrix elements are [24]

(21) $$\left\{\begin{array}{ll} v_{11} & =-p_{7}^{2}-q_{7}^{2}+p_{8}^{2}+q_{8}^{2}+p_{9}^{2}+q_{9}^{2}\\ & =\sum_{k=1}^{N}c_{k}^{2}\left(1-2m_{7k}^{2}\right)+\sum_{k,l=1;k\neq l}^{N}c_{k}c_{l}\left({\hat{m}}_{k}\cdot {\hat{m}}_{l}-2m_{7k}m_{7l}\right)\cos2∆\varphi_{kl}\\ v_{22} & =p_{7}^{2}+q_{7}^{2}-p_{8}^{2}-q_{8}^{2}+p_{9}^{2}+q_{9}^{2}\\ & =\sum_{k=1}^{N}c_{k}^{2}\left(1-2m_{8k}^{2}\right)+\sum_{k,l=1;k\neq l}^{N}c_{k}c_{l}\left({\hat{m}}_{k}\cdot {\hat{m}}_{l}-2m_{8k}m_{8l}\right)\cos2∆\varphi_{kl}\\ v_{33} & =p_{7}^{2}+q_{7}^{2}+p_{8}^{2}+q_{8}^{2}-p_{9}^{2}-q_{9}^{2}\\ & =\sum_{k=1}^{N}c_{k}^{2}\left(1-2m_{9k}^{2}\right)+\sum_{k,l=1;k\neq l}^{N}c_{k}c_{l}\left({\hat{m}}_{k}\cdot {\hat{m}}_{l}-2m_{9k}m_{9l}\right)\cos2∆\varphi_{kl}\\ v_{12} & =-2\left(p_{7}p_{8}+q_{7}q_{8}\right)\\ & =-2\sum_{k=1}^{N}c_{k}^{2}\left(m_{7k}m_{8k}\right)-2\sum_{k,l=1;k\neq l}^{N}c_{k}c_{l}\left(m_{7k}m_{8l}\right)\cos2∆\varphi_{kl}\\ v_{13} & =-2\left(p_{7}p_{9}+q_{7}q_{9}\right)\\ & =-2\sum_{k=1}^{N}c_{k}^{2}\left(m_{7k}m_{9k}\right)-2\sum_{k,l=1;k\neq l}^{N}c_{k}c_{l}\left(m_{7k}m_{9l}\right)\cos2∆\varphi_{kl}\;\\ v_{23} & =-2\left(p_{8}p_{9}+q_{8}q_{9}\right)\\ & =-2\sum_{k=1}^{N}c_{k}^{2}\left(m_{8k}m_{9k}\right)-2\sum_{k,l=1;k\neq l}^{N}c_{k}c_{l}\left(m_{8k}m_{9l}\right)\cos2∆\varphi_{kl} \end{array}\right.$$

The Mueller matrix $M_{RTS}$ in Equation (20) can also be decomposed into an orthogonal Mueller matrix $M_{U}$ representing birefringence and a symmetric Mueller matrix $M_{H}$ representing PDL as [29]

(22) $$M_{RTS}=M_{U}M_{H}$$

Two sub-matrices $M_{U}$ and $M_{H}$ can be computed using the method proposed in [29] or directly converted from the corresponding sub-Jones matrices U and H, respectively. The PDL–Mueller matrix $M_{H}$ is

(23) $$M_{H}=\left[\begin{array}{cc} \begin{array}{cc} h_{0}^{2}+h^{2} & 2h_{0}h_{1}\\ 2h_{0}h_{1} & h_{0}^{2}+2h_{1}^{2}-h^{2} \end{array} & \begin{array}{cc} 2h_{0}h_{2} & \;\;\;\;\;\;\;\;\;\;\;\;\;\;\;2h_{0}h_{3}\\ 2h_{1}h_{2} & \;\;\;\;\;\;\;\;\;\;\;\;\;\;\;2h_{1}h_{3} \end{array}\\ \begin{array}{cc} 2h_{0}h_{2} & \;\;\;\;\;\;\;\;\;\;2h_{1}h_{2}\;\;\;\;\;\;\\ 2h_{0}h_{3} & \;\;\;\;2h_{1}h_{3} \end{array} & \begin{array}{cc} h_{0}^{2}+2h_{2}^{2}-h^{2} & 2h_{2}h_{3}\\ 2h_{2}h_{3} & h_{0}^{2}+{2h}_{3}^{2}-h^{2} \end{array} \end{array}\right]$$

The definitions of h and $h_{j},\;j=0,1,2,3$ can be found in Equation (12). Here, more relationships are given:

(24) $$\left\{\begin{array}{l} h_{0}^{2}+h^{2}=w_{0},\;\;\;h_{0}^{2}-h^{2}=\sqrt{w_{0}^{2}-w^{2}}\\ 2h_{0}h_{j}=w_{j}\;\;\;\;j=1,2,3\;\;\;\;\;\;\;\;\;\;\;\;\;\;\;\;\;\;\;\;\;\;\;\;\;\;\;\;\;\;\;\;\;\;\;\;\;\;\\ 2h_{j}h_{k}=\frac{w_{j}w_{k}}{w_{0}+\sqrt{w_{0}^{2}-w^{2}}}\;\;\;j,k=1,2,3\;\;\;\;\;\;\;\; \end{array}\right.$$

With equations in Equations (12) and (24), $M_{H}$ can also be expressed as

(25) $$M_{H}=\left[\begin{array}{cccc} w_{0} & w_{1} & w_{2} & w_{3}\\ w_{1} & \sqrt{w_{0}^{2}-w^{2}}+\frac{w_{1}^{2}}{w_{0}+\sqrt{w_{0}^{2}-w^{2}}} & \frac{w_{1}w_{2}}{w_{0}+\sqrt{w_{0}^{2}-w^{2}}} & \frac{w_{1}w_{3}}{w_{0}+\sqrt{w_{0}^{2}-w^{2}}}\\ w_{2} & \frac{w_{1}w_{2}}{w_{0}+\sqrt{w_{0}^{2}-w^{2}}} & \sqrt{w_{0}^{2}-w^{2}}+\frac{w_{2}^{2}}{w_{0}+\sqrt{w_{0}^{2}-w^{2}}} & \frac{w_{2}w_{3}}{w_{0}+\sqrt{w_{0}^{2}-w^{2}}}\\ w_{3} & \frac{w_{1}w_{3}}{w_{0}+\sqrt{w_{0}^{2}-w^{2}}} & \frac{w_{2}w_{3}}{w_{0}+\sqrt{w_{0}^{2}-w^{2}}} & \sqrt{w_{0}^{2}-w^{2}}+\frac{w_{3}^{2}}{w_{0}+\sqrt{w_{0}^{2}-w^{2}}} \end{array}\right]$$

The birefringence Mueller matrix $M_{U}$ is

(26) $$M_{U}=\frac{\left[\begin{array}{cccc} {\left(h_{0}^{2}-h^{2}\right)}^{2} & 0 & 0 & 0\\ 0 & \eta_{11} & \eta_{12} & \eta_{13}\\ 0 & \eta_{21} & \eta_{22} & \eta_{23}\\ 0 & -\eta_{31} & -\eta_{32} & -\eta_{33} \end{array}\right]}{{\left(h_{0}^{2}-h^{2}\right)}^{2}}$$

where the factors $\eta_{jk},\;j,k=1,2,3$ are defined as

(27) $$\left\{\begin{array}{l} \eta_{11}=\left(h_{0}^{2}+2h_{1}^{2}-h^{2}\right)v_{11}-4h_{0}^{2}h_{1}^{2}+2h_{1}h_{2}v_{12}+2h_{1}h_{3}v_{13}\;\;\;\;\\ \eta_{22}=\left(h_{0}^{2}+2h_{2}^{2}-h^{2}\right)v_{22}-4h_{0}^{2}h_{2}^{2}+2h_{1}h_{2}v_{12}+2h_{2}h_{3}v_{23}\;\;\;\;\\ \eta_{33}=\left(h_{0}^{2}+2h_{3}^{2}-h^{2}\right)v_{33}-4h_{0}^{2}h_{3}^{2}+2h_{1}h_{3}v_{13}+2h_{2}h_{3}v_{23}\;\;\;\;\\ \eta_{12}=\left(h_{0}^{2}+2h_{2}^{2}-h^{2}\right)v_{12}-4h_{0}^{2}h_{1}h_{2}+2h_{1}h_{2}v_{11}+2h_{2}h_{3}v_{13}\\ \eta_{21}=\left(h_{0}^{2}+2h_{1}^{2}-h^{2}\right)v_{12}-4h_{0}^{2}h_{1}h_{2}+2h_{1}h_{2}v_{22}+2h_{1}h_{3}v_{23}\\ \eta_{13}=\left(h_{0}^{2}+2h_{3}^{2}-h^{2}\right)v_{13}-4h_{0}^{2}h_{1}h_{3}+2h_{1}h_{3}v_{11}+2h_{2}h_{3}v_{12}\\ \eta_{31}=\left(h_{0}^{2}+2h_{1}^{2}-h^{2}\right)v_{12}-4h_{0}^{2}h_{1}h_{3}+2h_{1}h_{2}v_{23}+2h_{1}h_{3}v_{33}\\ \eta_{23}=\left(h_{0}^{2}+2h_{3}^{2}-h^{2}\right)v_{23}-4h_{0}^{2}h_{2}h_{3}+2h_{1}h_{3}v_{12}+2h_{2}h_{3}v_{22}\\ \eta_{32}=\left(h_{0}^{2}+2h_{2}^{2}-h^{2}\right)v_{23}-4h_{0}^{2}h_{2}h_{3}+2h_{1}h_{2}v_{13}+2h_{2}h_{3}v_{33} \end{array}\right.$$

## 4. Phase and Intensity Measurement in φ-OTDR

**Figure 2:**
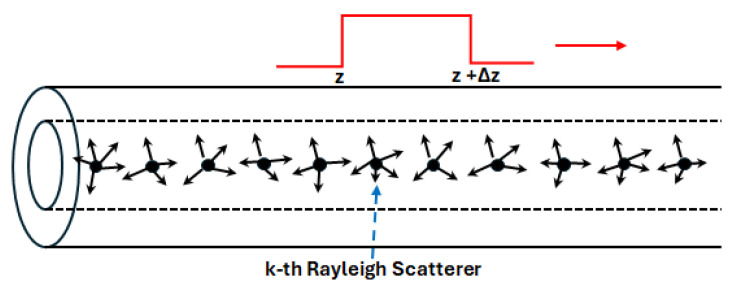
An optical pulse is travelling in the SMF. The pulse width is Δz. The falling edge and the leading edge of the optical pulse are at the fiber length z and z+Δz, respectively.

As discussed in [24], the derived formulas in Section 2 and Section 3 are rigorously valid when the incoming probe is a continuous wave (CW) light with an infinite long coherence length. In φ-OTDR, the coherent RB is generated by the incoming pulsed light with a finite coherence length, as shown in Figure 2. However, the derived formulas can still be applied to φ-OTDR to study its features in the sense of the first-order approximation. In this section, we investigate the properties of the common optical phase and the intensity (optical power) of the φ-OTDR signal using the polar decomposition results in Section 2 and Section 3.

When the optical pulse resides in the fiber section from z to z+Δz (Δz is the pulse width), only the Rayleigh scatterers in the fiber section from z to z+Δz/2 contribute to the coherent RB light corresponding to the fiber position z. If the sequence numbers of the Rayleigh scatterer at z and z+Δz/2 are denoted as $N_{z}$ and $N_{z+\Delta z/2}$, respectively, all derived formulas in Section 2 and Section 3 and Appendix A, Appendix B, Appendix C, Appendix D and Appendix E are still valid after changing the lower bound and upper bound of the summation from 1 and N to $N_{z}$ and $N_{z+\Delta z/2}$, respectively. Note that $1\leq N_{z}<N_{z+\Delta z/2}\leq N$. For instance, $w_{j},\;j=0,1,2,3$ in Equation (10) are expressed as

(28) $$\left\{\begin{array}{l} w_{0}\left(z\right)=\sum_{k,l=N_{z}}^{N_{z+\frac{\Delta z}{2}}}c_{k}c_{l}\left({\hat{m}}_{k}\cdot {\hat{m}}_{l}\right)\cos2∆\varphi_{kl}\\ w_{1}\left(z\right)=\sum_{k,l=N_{z}}^{N_{z+\frac{\Delta z}{2}}}c_{k}c_{l}\left(m_{8k}m_{9l}-m_{8l}m_{9k}\right)\sin2∆\varphi_{kl}\;\\ w_{2}\left(z\right)=\sum_{k,l=N_{z}}^{N_{z+\frac{\Delta z}{2}}}c_{k}c_{l}\left(m_{9k}m_{7l}-m_{9l}m_{7k}\right)\sin2∆\varphi_{kl}\;\\ w_{3}\left(z\right)=\sum_{k,l=N_{z}}^{N_{z+\frac{\Delta z}{2}}}c_{k}c_{l}\left(m_{7k}m_{8l}-m_{7l}m_{8k}\right)\sin2∆\varphi_{kl}\; \end{array}\right.$$

The notations in Equation (28) have been defined in the context from Equation (1) to Equation (10).

### 4.1. Optical Power

In φ-OTDR, the optical power corresponding to the fiber position z, based on Equation (20) or Equation (25), can be obtained as

(29) $$P\left(z\right)=S_{out0}\left(z\right)=\sum_{j=0}^{3}w_{j}\left(z\right)S_{inj}$$

where $\left[S_{in0}\;S_{in1}\;S_{in2}\;S_{in3}\right]$ is the input Stokes vector, representing the input SOP at the fiber input end. $w_{j}\left(z\right),\;j=0,1,2,3$ are in the forms of Equation (28).

From Equation (29), it can be noticed that $P\left(z\right)$ depends on the input SOP $\left[S_{in0}\;S_{in1}\;S_{in2}\;S_{in3}\right]$. When the input SOP is fixed, $P\left(z\right)$ is determined by $w_{j}\left(z\right),\;j=0,1,2,3$. From Equation (28), $w_{0}\left(z\right)$ is decided by ${\hat{m}}_{k}\cdot {\hat{m}}_{l}$ and $\cos2∆\varphi_{kl}$. Because $∆\varphi_{kl}$ is the phase difference between two Rayleigh scatterers in the fiber section from z to z+Δz/2, it is not affected by any phase variation in the fiber section before z and after z+Δz/2. Further, as demonstrated in Appendix E, ${\hat{m}}_{k}\cdot {\hat{m}}_{l}$ is also not affected by any birefringence variation in the fiber section before z and after z+Δz/2. Therefore, $w_{0}\left(z\right)$ is a local parameter that is only sensitive to the local phase and birefringence variations within the first half of the optical pulse. The experimental verification of this conclusion has been reported in [19].

However, $w_{j}\left(z\right),\;j=1,2,3$ are affected by the birefringence variation before the fiber position z because the direction of the vector ${\hat{m}}_{k}\cdot {\hat{m}}_{l}$ is rotated by the birefringence variation before z.

Further, from Equation (A8) in Appendix B, the parameter $w\left(z\right)=\sqrt{\sum_{j=1}^{3}{w_{j}}^{2}}$ is also a local parameter that is immune to the birefringence variation and the phase variation before z and after z+Δz/2. Since $w_{0}\left(z\right)$ and $w\left(z\right)$ are local parameters, the *PDL*, which is defined as $PDL\left(dB\right)=10\log_{10}\frac{w_{0}+w}{w_{0}-w}$ [30], is also a local parameter.

To measure $w_{0}\left(z\right)$ in φ-OTDR, two optical power measurements $P_{1}\left(z\right)$ and $P_{2}\left(z\right)$ need to be performed with two orthogonal input SOPs $\left[S_{in0}\;S_{in1}\;S_{in2}\;S_{in3}\right]$ and $\left[S_{in0}-S_{in1}-S_{in2}-S_{in3}\right]$, respectively [19]. Then, $w_{0}\left(z\right)$ can be calculated from

(30) $$w_{0}\left(z\right)=\frac{P_{1}\left(z\right)+P_{2}\left(z\right)}{2S_{in0}}$$

To measure $w\left(z\right)$ or PDL in φ-OTDR, four optical power measurements $P_{j}\left(z\right),\;j=1,2,3,4$ need to be performed with four different SOPs $\left[1\;1\;0\;0\right]$, $\left[1-1\;0\;0\right]$, $\left[1\;0\;1\;0\right]$, and $\left[1\;0\;0\;1\right]$, respectively [30]. Then, it has

(31) $$\left\{\begin{array}{l} w_{0}\left(z\right)=\frac{P_{1}\left(z\right)+P_{2}\left(z\right)}{2}\;\;\;\;\;\;\;\;\;\;\;\;\;\;\;\;\;\;\;\;\;\;\;\;\;\;\;\;\;\;\;\;\;\;\;\;\;\;\;\;\;\;\;\;\;\;\;\;\;\;\;\;\;\;\;\;\;\;\;\;\;\;\;\;\;\\ w\left(z\right)=\frac{\sqrt{{\left(P_{1}-P_{2}\right)}^{2}+{\left(P_{3}-P_{1}-P_{2}\right)}^{2}+{\left(P_{4}-P_{1}-P_{2}\right)}^{2}}}{2} \end{array}\right.$$

If the perturbations applied to the SMF are relatively weak, for instance, the dynamic strain is 1 με [19], so that only the optical phase variations are obvious, and the birefringence variations can be neglected, $w_{j}\left(z\right),\;j=0,1,2,3$ can be local parameters. In this case, $P\left(z\right)$ can also be considered as a local parameter, which is why the intensity-based φ-OTDR can realize distributed vibration sensing. The dependency of the parameters to the variations in the birefringence and the optical phase before z is summarized in Table 1.

### 4.2. Optical Phase

The optical phase of the φ-OTDR signal is of great importance because it is used to calculate the local strain variation [28]. When the optical pulse location is from z to z+Δz, the common optical phase $\varepsilon \left(z\right)$ measured at the fiber input end should be

(32) $$\varepsilon \left(z\right)=2\varphi_{z}-\frac{\tau \left(z\right)}{2}=2\varphi_{z}-\frac{\tan^{-1}\left[\frac{\sum_{k,l=N_{z}}^{N_{z+\frac{∆z}{2}}}c_{k}c_{l}\left({\hat{m}}_{k}\cdot {\hat{m}}_{l}\right)\sin2\left(∆\varphi_{k}+∆\varphi_{l}\right)}{\sum_{k,l=N_{z}}^{N_{z+\frac{∆z}{2}}}c_{k}c_{l}\left({\hat{m}}_{k}\cdot {\hat{m}}_{l}\right)\cos2\left(∆\varphi_{k}+∆\varphi_{l}\right)}\right]}{2}$$

where $∆\varphi_{k}=\varphi_{k}-\varphi_{z}$ and $∆\varphi_{l}=\varphi_{l}-\varphi_{z}$. $\varphi_{z}$ is the optical phase at the fiber position z. It is evident that $\tau \left(z\right)$ is a local parameter, which is completely determined by the optical phase distribution $∆\varphi_{k}+∆\varphi_{l}$ and the birefringence distribution ${\hat{m}}_{k}\cdot {\hat{m}}_{l}$ within the first half of the optical pulse.

In a phase-based φ-OTDR, we usually measure the optical phases at two adjacent fiber locations $z_{1}$ and $z_{2}$ at two neighboring times $T_{1}$ and $T_{2}$, then the phase difference is calculated as [28]

(33) $$∆\varphi =\left[\varepsilon \left(z_{2},\;T_{2}\right)-\varepsilon \left(z_{1},\;T_{2}\right)\right]-\left[\varepsilon \left(z_{2},\;T_{1}\right)-\varepsilon \left(z_{1},\;T_{1}\right)\right]$$

If a perturbation is happening in the fiber section from $z_{1}+∆z/2$ to $z_{2}$, it has

(34) $$\tau \left(z_{2},T_{2}\right)=\tau \left(z_{2},T_{1}\right),\;\tau \left(z_{1},T_{2}\right)=\tau \left(z_{1},T_{1}\right)$$

With Equation (34), Equation (33) becomes

(35) $$∆\varphi =2\left\{\left[\varphi_{z_{2}}\left(T_{2}\right)-\varphi_{z_{1}}\left(T_{2}\right)\right]-\left[\varphi_{z_{2}}\left(T_{1}\right)-\varphi_{z_{1}}\left(T_{1}\right)\right]\right\}$$

Different from the measurement of the optical power in φ-OTDR, even the strong perturbations, which can alter the fiber birefringence, will not affect the measurement of ∆φ as long as two fiber sections are not disturbed—one is from $z_{1}$ to $z_{1}+\Delta z/2$, the other is $z_{2}$ to $z_{2}+\Delta z/2$. Moreover, ∆φ is also not affected by the variation in the input SOP.

## 5. Conclusions

Polar decompositions of the Jones matrix and the Mueller matrix of the coherent RB in SMFs are performed. The derived sub-matrices reveal the relationships between the polarization properties of the coherent RB and the distributions of the optical phase and fiber birefringence. These theoretical formulas can be used to investigate the influences of the coherent RB in fiber-optic communication and sensing systems. As an application of the derived sub-matrices in φ-OTDR, they are used to investigate the polarization properties of the common optical phase and the intensity of the φ-OTDR signal. It is theoretically demonstrated, in φ-OTDR, that (1) the common optical phase is immune from the input SOP variation and the birefringence variation in the fiber section before the probing pulse position; (2) the intensity is affected by the input SOP and the birefringence variation in the fiber section before the pulse position, like the case in polarization-OTDR. However, when the vibrations applied to SMFs are weak, for example, 1 με [19], the intensity can be still considered as a local parameter for distributed vibration sensing; (3) two parameters, *w*_0_ and PDL, are not affected by the input SOP variation and the birefringence variation in the fiber section before the pulse position, which are local parameters that can be measured through a polarimetric φ-OTDR. To confirm the existence of the PDL effect in the coherent RB, an experiment is performed using a short SMF. The experimental configuration and results are shown in Appendix F.

## Figures and Tables

**Table 1:** The dependency of the parameters to the variations in the birefringence and the optical phase before z.

	P	$w_{0}$	$w_{j},j=1,2,3$	w	PDL	ε
Birefringence	Yes	No	Yes	No	No	No
Phase	No	No	No	No	No	Yes

**Figure A1:**
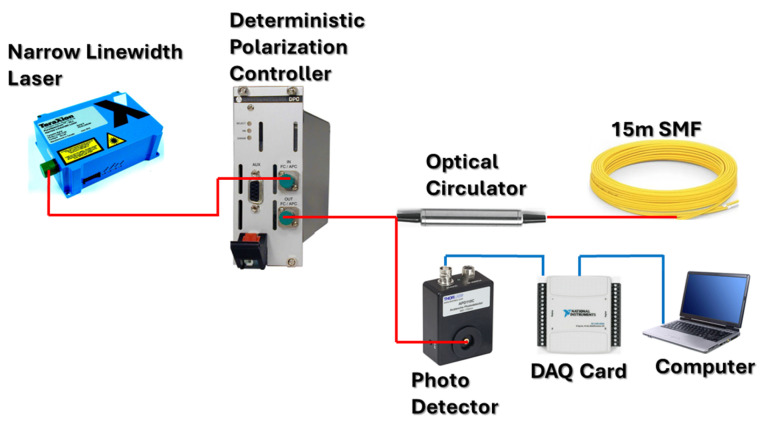
The experimental setup to measure the PDL of coherent RB using a CW light and a 15 m long SMF. DAQ card: data acquisition card.

**Figure A2:**
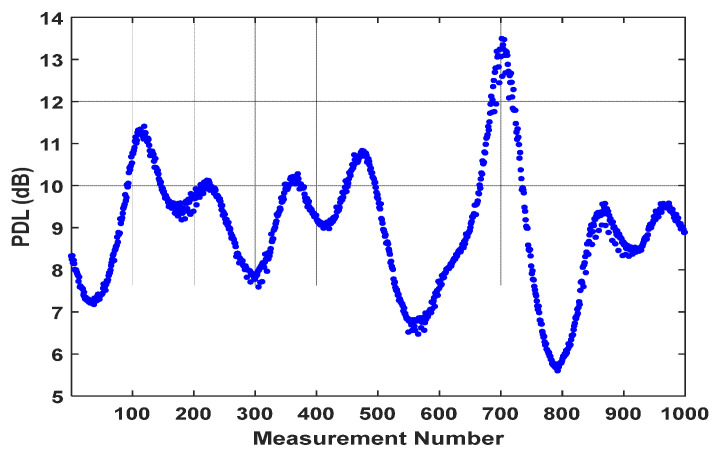
The measurement results of PDL values in 1000 independent measurements using a CW light and a 15 m long SMF.

## Data Availability

The data in this work are available from the corresponding author upon request.

## Appendix A

As shown in Figure 1, the Mueller matrix M depicts the polarization properties of the fiber section from the SMF input end to the *k*-th Rayleigh scatterer. As the SMF is purely birefringent, M can be expressed as

(A1) $$M=e^{-\rho }\left[\begin{array}{cccc} 1 & 0 & 0 & 0\\ 0 & m_{1} & m_{2} & m_{3}\\ 0 & m_{4} & m_{5} & m_{6}\\ 0 & m_{7} & m_{8} & m_{9} \end{array}\right]$$

where ρ stands for fiber attenuation. Because $e^{\rho }M$ is an orthogonal matrix, the sub-matrix elements can be written as those of a birefringent element (retarder) [31]

(A2) $$\left[\begin{array}{ccc} m_{1} & m_{2} & m_{3}\\ m_{4} & m_{5} & m_{6}\\ m_{7} & m_{8} & m_{9} \end{array}\right]=\cos\delta \cdot I+\left(1-\cos\delta \right)\hat{r}\hat{r}-\sin\delta \cdot \hat{r}\times$$

where δ is the retardance of the retarder; I is the 3×3 identity matrix; $\hat{r}=\left[r_{1}\;r_{2}\;r_{3}\right]$ is a unit vector, representing the fast axis of the retarder in Stokes space; $\hat{r}\hat{r}$ is a dyadic, and $\hat{r}\times$ is the cross-product operator. To highlight this, three matrix elements at the fourth row are

(A3) $$\left\{\begin{array}{l} m_{7}=\left(1-\cos\delta \right)r_{1}r_{3}+r_{2}\sin\delta \\ m_{8}=\left(1-\cos\delta \right)r_{2}r_{3}-r_{1}\sin\delta \\ m_{9}=\cos\delta +\left(1-\cos\delta \right)r_{3}^{2}\;\;\;\;\;\; \end{array}\right.$$

On the other hand, these Mueller matrix elements can also be expressed in terms of three Euler angles ψ, θ, and ϕ in the form [32]

(A4) $$\left[\begin{array}{ccc} m_{1} & m_{2} & m_{3}\\ m_{4} & m_{5} & m_{6}\\ m_{7} & m_{8} & m_{9} \end{array}\right]\;=\left[\begin{array}{ccc} \cos\theta \cos\phi & \sin\psi \sin\theta \cos\phi -\cos\psi \sin\phi & \cos\psi \sin\theta \cos\phi +\sin\psi \sin\phi \\ \cos\theta \sin\phi & \sin\psi \sin\theta \sin\phi +\cos\psi \cos\phi & \cos\psi \sin\theta \sin\phi -\sin\psi \cos\phi \\ -\sin\theta & \sin\psi \cos\theta \;\; & \cos\psi \cos\theta \end{array}\right]$$

Three Euler angles ψ, θ, and ϕ denote the rotation angles about the $s_{1}$-axis, the $s_{2}$-axis, and the $s_{3}$-axis in the Stokes space, respectively. Using three Euler angles, three matrix elements at the fourth row can be expressed in a simpler form, compared to Equation (A3).

(A5) $$\left\{\begin{array}{l} m_{7}=-\sin\theta \\ m_{8}=\sin\psi \cos\theta \\ m_{9}=\cos\psi \cos\theta \end{array}\right.$$

## Appendix B

From Equations (3) and (10), it can be obtained that

(A6) $$\vec{q}\times \vec{p}=\frac{\left[w_{1}\;\;w_{2}\;\;w_{3}\right]}{2}$$

Based on the well-known mathematical inequalities, it has

(A7) $$w_{0}={\left|\vec{p}\right|}^{2}+{\left|\vec{q}\right|}^{2}\geq 2\left|\vec{p}\right|\cdot \left|\vec{q}\right|\geq 2\left|\vec{q}\times \vec{p}\right|=w=\sqrt{\sum_{j=1}^{3}w_{j}^{2}}$$

w can also be expressed in terms of the optical phase and the elements of the forward Mueller matrix. It has

(A8) $$\begin{array}{l} w=\sqrt{\sum_{j=1}^{3}{w_{j}}^{2}}\\ =\sqrt{\sum_{k,l,g,j=1}^{N}c_{k}c_{l}c_{g}c_{j}\left[\begin{array}{c} \left(m_{8k}m_{9l}-m_{8l}m_{9k}\right)\left(m_{8g}m_{9j}-m_{8j}m_{9g}\right)+\\ \left(m_{9k}m_{7l}-m_{9l}m_{7k}\right)\left(m_{9g}m_{7j}-m_{9j}m_{7g}\right)+\\ \left(m_{7k}m_{8l}-m_{7l}m_{8k}\right)\left(m_{7g}m_{8j}-m_{7j}m_{8g}\right) \end{array}\right]\sin2∆\varphi_{kl}\sin2∆\varphi_{gj}\;}\\ =\sqrt{\sum_{k,l,g,j=1}^{N}c_{k}c_{l}c_{g}c_{j}\left[\left({\hat{m}}_{k}\cdot {\hat{m}}_{l}\right)\cdot \left({\hat{m}}_{g}\cdot {\hat{m}}_{j}\right)\right]\sin2∆\varphi_{kl}\sin2∆\varphi_{gj}\;}\\ =\sqrt{\sum_{k,l,g,j=1}^{N}c_{k}c_{l}c_{g}c_{j}\left[\left({\hat{m}}_{k}\cdot {\hat{m}}_{g}\right)\left({\hat{m}}_{l}\cdot {\hat{m}}_{j}\right)-\left({\hat{m}}_{k}\cdot {\hat{m}}_{j}\right)\left({\hat{m}}_{l}\cdot {\hat{m}}_{g}\right)\right]\sin2∆\varphi_{kl}\sin2∆\varphi_{gj}\;} \end{array}$$

To obtain Equation (A8), we used the vector equation $\left(\vec{a}\times \vec{b}\right)\cdot \left(\vec{c}\times \vec{d}\right)=\left(\vec{a}\cdot \vec{c}\right)\left(\vec{b}\cdot \vec{d}\right)-\left(\vec{a}\cdot \vec{d}\right)\left(\vec{b}\cdot \vec{c}\right)$.

The difference between $w_{0}^{2}$ and $w^{2}$ is calculated and shown below, as follows:

(A9) $$w_{0}^{2}-w^{2}={\left(p_{7}^{2}+p_{8}^{2}+p_{9}^{2}-q_{7}^{2}-q_{8}^{2}-q_{9}^{2}\right)}^{2}+4{\left(p_{7}q_{7}+p_{8}q_{8}+p_{9}q_{9}\right)}^{2}\;={\left[\sum_{k,l=1}^{N}c_{k}c_{l}\left({\hat{m}}_{k}\cdot {\hat{m}}_{l}\right)\cos2\left(\varphi_{k}+\varphi_{l}\right)\;\right]}^{2}+{\left[\sum_{k,l=1}^{N}c_{k}c_{l}\left({\hat{m}}_{k}\cdot {\hat{m}}_{l}\right)\sin2\left(\varphi_{k}+\varphi_{l}\right)\;\right]}^{2}\;=\sum_{k,l,g,j=1}^{N}c_{k}c_{l}c_{g}c_{j}\left({\hat{m}}_{k}\cdot {\hat{m}}_{l}\right)\left({\hat{m}}_{g}\cdot {\hat{m}}_{j}\right)\cos2\left(∆\varphi_{kg}+∆\varphi_{lj}\right)\;$$

## Appendix C

From Equation (14), the determinant of U is

(A10) $$det\left(U\right)=det\left(J_{RTS}H^{-1}\right)=det\left(J_{RTS}\right)\cdot det\left(H^{-1}\right)$$

From Equation (1), the determinant of $J_{RTS}$ is

(A11) $$det\left(J_{RTS}\right)=t_{1}t_{3}-t_{2}^{2}$$

From Equation (13), the determinant of $H^{-1}$ is

(A12) $$det\left(H^{-1}\right)=\frac{\left(h_{0}-h_{1}\right)\left(h_{0}+h_{1}\right)-\left(h_{2}-ih_{3}\right)\left(h_{2}+ih_{3}\right)}{{\left(h_{0}^{2}-h^{2}\right)}^{2}}=\frac{h_{0}^{2}-h_{1}^{2}-h_{2}^{2}-h_{3}^{2}}{{\left(h_{0}^{2}-h^{2}\right)}^{2}}=\frac{1}{h_{0}^{2}-h^{2}}$$

Therefore, from the above three equations, it has

(A13) $$det\left(U\right)=\frac{t_{1}t_{3}-t_{2}^{2}}{h_{0}^{2}-h^{2}}$$

From Equation (2), the numerator of Equation (A13) is

(A14) $$t_{1}t_{3}-t_{2}^{2}=\left[q_{8}+p_{9}+i\left(q_{9}-p_{8}\right)\right]\left[-q_{8}+p_{9}+i\left(q_{9}+p_{8}\right)\right]-{\left(-q_{7}+ip_{7}\right)}^{2}\;=p_{7}^{2}+p_{8}^{2}+p_{9}^{2}-q_{7}^{2}-q_{8}^{2}-q_{9}^{2}+2i\left(p_{7}q_{7}+p_{8}q_{8}+p_{9}q_{9}\right)$$

From Equations (10) and (12), the denominator of Equation (A13) is

(A15) $$h_{0}^{2}-h^{2}=\sqrt{w_{0}^{2}-w^{2}}=\sqrt{{\left(p_{7}^{2}+p_{8}^{2}+p_{9}^{2}-q_{7}^{2}-q_{8}^{2}-q_{9}^{2}\right)}^{2}+4{\left(p_{7}q_{7}+p_{8}q_{8}+p_{9}q_{9}\right)}^{2}}$$

According to Equations (A14) and (A15), it is obvious that the modulus of $det\left(U\right)$ is

(A16) $$\left|det\left(U\right)\right|=\frac{\left|t_{1}t_{3}-t_{2}^{2}\right|}{h_{0}^{2}-h^{2}}=1$$

Hence, $det\left(U\right)$ can be expressed in the form of

(A17) $$det\left(U\right)=e^{i\tau }$$

Using the real part and the imaginary part of Equation (A14), the angle τ can be calculated from

(A18) $$tan\tau =\frac{2\left(p_{7}q_{7}+p_{8}q_{8}+p_{9}q_{9}\right)}{p_{7}^{2}+p_{8}^{2}+p_{9}^{2}-q_{7}^{2}-q_{8}^{2}-q_{9}^{2}}$$

From Equation (3), it can be obtained that

(A19) $$\begin{array}{l} 2\left(p_{7}q_{7}+p_{8}q_{8}+p_{9}q_{9}\right)=2\sum_{j=7}^{9}\left[\left(\sum_{k=1}^{N}c_{k}m_{jk}\cos2\varphi_{k}\right)\left(\sum_{l=1}^{N}c_{l}m_{jl}\sin2\varphi_{l}\right)\right]\\ \;=2\sum_{k,l=1}^{N}c_{k}c_{l}\cos2\varphi_{k}\sin2\varphi_{l}\left(m_{7k}m_{7l}+m_{8k}m_{8l}+m_{9k}m_{9l}\right)\\ \;=2\sum_{k,l=1}^{N}c_{k}c_{l}\cos2\varphi_{k}\sin2\varphi_{l}\left({\hat{m}}_{k}\cdot {\hat{m}}_{l}\right)\\ \;=\sum_{k,l=1}^{N}c_{k}c_{l}\left(\cos2\varphi_{k}\sin2\varphi_{l}+\cos2\varphi_{l}\sin2\varphi_{k}\right)\left({\hat{m}}_{k}\cdot {\hat{m}}_{l}\right)\\ \;=\sum_{k,l=1}^{N}c_{k}c_{l}\left({\hat{m}}_{k}\cdot {\hat{m}}_{l}\right)\sin2\left(\varphi_{k}+\varphi_{l}\right) \end{array}$$

(A20) $$\begin{array}{l} p_{7}^{2}+p_{8}^{2}+p_{9}^{2}-q_{7}^{2}-q_{8}^{2}-q_{9}^{2}\\ \;=\sum_{k,l=1}^{N}c_{k}c_{l}\cos2\varphi_{k}\cos2\varphi_{l}\left(m_{7k}m_{7l}+m_{8k}m_{8l}+m_{9k}m_{9l}\right)\\ \;-\sum_{k,l=1}^{N}c_{k}c_{l}\sin2\varphi_{k}\sin2\varphi_{l}\left(m_{7k}m_{7l}+m_{8k}m_{8l}+m_{9k}m_{9l}\right)\\ \;=\sum_{k,l=1}^{N}c_{k}c_{l}\left(\cos2\varphi_{k}\cos2\varphi_{l}-\sin2\varphi_{k}\sin2\varphi_{l}\right)\left({\hat{m}}_{k}\cdot {\hat{m}}_{l}\right)\\ \;=\sum_{k,l=1}^{N}c_{k}c_{l}\left({\hat{m}}_{k}\cdot {\hat{m}}_{l}\right)\cos2\left(\varphi_{k}+\varphi_{l}\right) \end{array}$$

Finally, it has

(A21) $$tan\tau =\frac{\sum_{k,l=1}^{N}c_{k}c_{l}\left({\hat{m}}_{k}\cdot {\hat{m}}_{l}\right)\sin2\left(\varphi_{k}+\varphi_{l}\right)}{\sum_{k,l=1}^{N}c_{k}c_{l}\left({\hat{m}}_{k}\cdot {\hat{m}}_{l}\right)\cos2\left(\varphi_{k}+\varphi_{l}\right)}$$

## Appendix D

When there is no birefringence effect in the SMF, it has

(A22) $${\hat{m}}_{k}=\left[0\;\;\;0\;\;\;1\right],\;k=1,\cdots ,\;N$$

Then, Equation (18) is simplified to

(A23) $$tan\tau =\frac{\sum_{k,l=1}^{N}c_{k}c_{l}\sin2\left(\varphi_{k}+\varphi_{l}\right)}{\sum_{k,l=1}^{N}c_{k}c_{l}\cos2\left(\varphi_{k}+\varphi_{l}\right)}$$

On the other hand, in this case, the electrical field of the coherent RB can be calculated as

(A24) $$E=\left|E\right|e^{i\varepsilon }=\sum_{k=1}^{N}c_{k}e^{2i\varphi_{k}}=\sum_{k=1}^{N}c_{k}\left(\cos{2\varphi }_{k}+i\sin{2\varphi }_{k}\right)\;=\sum_{k=1}^{N}c_{k}\cos{2\varphi }_{k}+i\sum_{k=1}^{N}c_{k}\sin2\varphi_{k}$$

Then, the optical phase ε is

(A25) $$\tan\varepsilon =\frac{\sum_{k=1}^{N}c_{k}\sin2\varphi_{k}}{\sum_{k=1}^{N}c_{k}\cos2\varphi_{k}}$$

In the next step, the following equation is calculated as follows:

(A26) $$\tan2\varepsilon =\frac{2\tan\varepsilon }{1-\tan^{2}\varepsilon }=\frac{2\frac{\sum_{k=1}^{N}c_{k}\sin2\varphi_{k}}{\sum_{k=1}^{N}c_{k}\cos2\varphi_{k}}}{1-\frac{\sum_{k=1}^{N}c_{k}\sin2\varphi_{k}}{\sum_{k=1}^{N}c_{k}\cos2\varphi_{k}}\cdot \frac{\sum_{l=1}^{N}c_{l}\sin2\varphi_{l}}{\sum_{l=1}^{N}c_{l}\cos2\varphi_{l}}}=\;\frac{2\left(\sum_{k=1}^{N}c_{k}\sin2\varphi_{k}\right)\left(\sum_{l=1}^{N}c_{l}\cos2\varphi_{l}\right)}{\left(\sum_{k=1}^{N}c_{k}\cos2\varphi_{k}\right)\left(\sum_{l=1}^{N}c_{l}\cos2\varphi_{l}\right)-\left(\sum_{k=1}^{N}c_{k}\sin2\varphi_{k}\right)\left(\sum_{l=1}^{N}c_{l}\sin2\varphi_{l}\right)}=\;\frac{\sum_{k,l=1}^{N}c_{k}c_{l}\left(\sin2\varphi_{k}\cos2\varphi_{l}+\sin2\varphi_{l}\cos2\varphi_{k}\right)}{\sum_{k,l=1}^{N}c_{k}c_{l}\left(\cos2\varphi_{k}\cos2\varphi_{l}-\sin2\varphi_{k}\sin2\varphi_{l}\right)}=\frac{\sum_{k,l=1}^{N}c_{k}c_{l}\sin2\left(\varphi_{k}+\varphi_{l}\right)}{\sum_{k,l=1}^{N}c_{k}c_{l}\cos2\left(\varphi_{k}+\varphi_{l}\right)}$$

Comparing Equation (A26) with Equation (A23), it also has τ=2ε. Hence, Equation (18) can be simplified to the known optical phase when the fiber birefringence is not considered. It also confirms the correctness of Equation (18).

## Appendix E

In this Appendix, we demonstrate that ${\hat{m}}_{k}\cdot {\hat{m}}_{l},\;k,\;l=1,\cdots ,\;N$ is solely determined by the local birefringence between the *k*-th Rayleigh scatterer and the *l*-th Rayleigh scatterer. In this case, we only need to use the sub-Mueller matrix of the forward Mueller matrix M in Equation (A1) for calculation. The forward sub-Mueller matrices at the *k*-th Rayleigh scatterer and the l-th Rayleigh scatterer are, respectively,

(A27) $$m_{k}=\left[\begin{array}{c} m_{1k}\;\;\;m_{2k}\;\;\;m_{3k}\\ m_{4k}\;\;\;m_{5k}\;\;\;m_{6k}\\ m_{7k}\;\;\;m_{8k}\;\;\;m_{9k} \end{array}\right]$$

(A28) $$m_{l}=\left[\begin{array}{c} m_{1l}\;\;\;m_{2l}\;\;\;m_{3l}\\ m_{4l}\;\;\;m_{5l}\;\;\;m_{6l}\\ m_{7l}\;\;\;m_{8l}\;\;\;m_{9l} \end{array}\right]$$

Because the SMF is purely birefringent, it has

(A29) $$m_{k}m_{k}^{T}=m_{k}^{T}m_{k}=I$$

where the subscript “T” denotes the matrix transpose.

The forward sub-Mueller matrix of the fiber section from the *k*-th Rayleigh scatterer to the l-th Rayleigh scatterer can be expressed as

(A30) $$m_{kl}=\left[\begin{array}{c} m_{1kl}\;\;\;m_{2kl}\;\;\;m_{3kl}\\ m_{4kl}\;\;\;m_{5kl}\;\;\;m_{6kl}\\ m_{7kl}\;\;\;m_{8kl}\;\;\;m_{9kl} \end{array}\right]$$

It also has

(A31) $$m_{l}=m_{kl}m_{k}$$

Then, the following equations can be obtained:

(A32) $$\left\{\begin{array}{l} m_{7l}=m_{7kl}m_{1k}+m_{8kl}m_{4k}+m_{9kl}m_{7k}\\ m_{8l}=m_{7kl}m_{2k}+m_{8kl}m_{5k}+m_{9kl}m_{8k}\\ m_{9l}=m_{7kl}m_{3k}+m_{8kl}m_{6k}+m_{9kl}m_{9k} \end{array}\right.$$

With Equation (A32), it has

(A33) $${\hat{m}}_{k}\cdot {\hat{m}}_{l}=m_{7k}m_{7l}+m_{8k}m_{8l}+m_{9k}m_{9l}\;=m_{7k}\left(m_{7kl}m_{1k}+m_{8kl}m_{4k}+m_{9kl}m_{7k}\right)+\;m_{8k}\left(m_{7kl}m_{2k}+m_{8kl}m_{5k}+m_{9kl}m_{8k}\right)+\;m_{9k}\left(m_{7kl}m_{3k}+m_{8kl}m_{6k}+m_{9kl}m_{9k}\right)\;=m_{7kl}\left(m_{1k}m_{7k}+m_{2k}m_{8k}+m_{3k}m_{9k}\right)+\;m_{8kl}\left(m_{4k}m_{7k}+m_{5k}m_{8k}+m_{6k}m_{9k}\right)+\;m_{9kl}\left(m_{7k}^{2}+m_{8k}^{2}+m_{9k}^{2}\right)\;\;\;\;\;\;\;\;\;\;\;\;\;\;\;\;\;\;\;\;\;\;\;\;=m_{9kl}=\cos\psi_{kl}\cos\theta_{kl}\;\;\;\;\;\;\;\;\;\;\;\;\;\;\;\;\;\;\;\;\;\;\;\;\;\;\;\;$$

Note that Equations (A5) and (A29) have been used to achieve Equation (A33). Equation (A33) means that ${\hat{m}}_{k}\cdot {\hat{m}}_{l},\;k,\;l=1,\cdots ,\;N$ only depends on the element $m_{9kl}$ of the local Mueller matrix $m_{kl}$.

Using Equations (7), (10) and (18) can be written in the new forms of

(A34) $$w_{0}=\sum_{k,l=1}^{N}c_{k}c_{l}m_{9kl}\cos2∆\varphi_{kl}\;=\sum_{k,l=1}^{N}c_{k}c_{l}\cos\psi_{kl}\cos\theta_{kl}\cos2∆\varphi_{kl}\;$$

(A35) $$tan\tau =\frac{\sum_{k,l=1}^{N}c_{k}c_{l}m_{9kl}\sin2\left(\varphi_{k}+\varphi_{l}\right)}{\sum_{k,l=1}^{N}c_{k}c_{l}m_{9kl}\cos2\left(\varphi_{k}+\varphi_{l}\right)}=\frac{\sum_{k,l=1}^{N}c_{k}c_{l}\cos\psi_{kl}\cos\theta_{kl}\sin2\left(\varphi_{k}+\varphi_{l}\right)}{\sum_{k,l=1}^{N}c_{k}c_{l}\cos\psi_{kl}\cos\theta_{kl}\cos2\left(\varphi_{k}+\varphi_{l}\right)}$$

## Appendix F

To verify the theoretical prediction that the coherent RB has a PDL effect in a purely birefringent SMF, an experiment is performed. In the experiment, a CW light is used to sense a short SMF. The experimental setup is plotted in Figure A1.

The linewidth of the narrow linewidth laser (NLL) used in the experiment is 1.8 kHz and the output CW power is 50 mW. This means that the coherence length of the NLL is greater than 100 km. The deterministic polarization controller (DPC) is an SOP scanner that can generate 1666 uniformly distributed SOP points on a 5-degree grid on the Poincaré sphere within 0.28 s. The PDL of the optical circulator (OC) is 0.04 dB from port 1 to port 2, and 0.02 dB from port 2 to port 3, which are negligible compared to the PDL under measurement. The fiber under test is a 15 m long standard SMF; its proximal and distal ends are properly treated to eliminate Fresnel reflections. In the first step, the DPC is not working and port 3 of the OC is connected to an optical spectrum analyzer. From the measured optical spectrum, it can be confirmed that the stimulated Brillouin scattering (SBS) light is negligible compared to the coherent RB light. Then, port 3 of the OC is connected to the photodetector and the DPC starts the SOP scans which are triggered and controlled by a developed computer program. During each SOP scan, the computer program records the optical power evolution P. When an SOP scan is completed, the PDL can be calculated using the maximum and minimum readings of P, i.e., $PDL\;\left(dB\right)=10lg\frac{P_{max}}{P_{min}}$. In the measurement, the SOP scans are repeated continuously for 1000 cycles; the 1000 measured PDL values are shown in Figure A2. The measurement results confirm that the coherent RB in an SMF has an intrinsic PDL effect. From Equation (10), the PDL value is jointly determined by the optical phase differences between Rayleigh backscatters and the fiber birefringence distribution. Since both optical phase and fiber birefringence are sensitive to ambient temperatures and vibrations, the measured PDL values also vary with time, as shown in Figure A2.
